# Predation Life History Responses to Increased Temperature Variability

**DOI:** 10.1371/journal.pone.0107971

**Published:** 2014-09-24

**Authors:** Miguel Barbosa, Joao Pestana, Amadeu M. V. M. Soares

**Affiliations:** 1 Center for Environmental and Marine Studies (CESAM), Departamento de Biologia, Universidade de Aveiro, Campus de Santiago, Aveiro, Portugal; 2 Centre for Biological Diversity and Scottish Oceans Institute, University of St Andrews, St Andrews, Fife, United Kingdom; 3 Programa de Pós-Graduação em Produção Vegetal, Universidade Federal de Tocantins, Campus de Gurupi, Gurupi, Brasil; University of Western Sydney, Australia

## Abstract

The evolution of life history traits is regulated by energy expenditure, which is, in turn, governed by temperature. The forecasted increase in temperature variability is expected to impose greater stress to organisms, in turn influencing the balance of energy expenditure and consequently life history responses. Here we examine how increased temperature variability affects life history responses to predation. Individuals reared under constant temperatures responded to different levels of predation risk as appropriate: namely, by producing greater number of neonates of smaller sizes and reducing the time to first brood. In contrast, we detected no response to predation regime when temperature was more variable. In addition, population growth rate was slowest among individuals reared under variable temperatures. Increased temperature variability also affected the development of inducible defenses. The combined effects of failing to respond to predation risk, slower growth rate and the miss-match development of morphological defenses supports suggestions that increased variability in temperature poses a greater risk for species adaptation than that posed by a mean shift in temperature.

## Introduction

Temperature directly affects metabolic rate and consequently energy expenditure. As a result, changes in temperature are often accompanied by both physiological and behavioural responses. One example of temperature change stress-induced disruption is the loss of the ability to recognize and respond to predation threat [1]. Recent global change data reveal that the environment is changing at an unprecedented pace, with temperature predicted to become increasingly stochastic [2]–[4]. While organisms are typically able to cope with a natural rate of temperature change, increased variability in temperature is likely to impose additional physiological stress, potentially affecting the way organisms respond to environmental conditions. Here we address this issue by examining the effects of increased variation in temperature on life history responses to predation risk.

The effects of predation risk on prey life history traits have been well established [5]–[7]. Under high predation risk, selection favours the production of more and smaller sized offspring, and fewer, bigger offspring when predation risk is low. Predation risk also induces responses in terms of the onset of reproduction. Generally, under high predation risk individuals mature and start reproducing sooner [8], [9]. Nevertheless, despite these life history expectations, the optimal reproductive response to predation risk is expected to be dynamic and primarily determined by energetic constraints [10].

Both predation risk and thermoregulation have associated energetic costs. The costs incurred by increased predation risk [11] can, together with thermoregulation requirements, interact and lead to a situation of greater stress, which can ultimately affect optimal life-history strategies. Further, individuals have a finite amount of energy to invest between growth, reproduction and maintenance [12]. Increased variability in mean temperatures, as predicted by global change models, are likely to influence how much energy is allocated to growth and thermoregulation. Specifically, under such circumstances of variability, organisms may be required to allocate more energy towards maintenance, because of thermoregulation, at the expense of growth or reproduction [13]. There is, therefore, the potential for a conflict between the energetic costs of thermoregulation and the energetic costs of life history responses to predation level.

Here we test the hypothesis that optimal life history responses to predation are impaired by increased variation in temperature using the waterflea *Daphnia magna*. Numerous studies have demonstrated that predation risk is an important driver of *Daphnia spp* life history [14]. Namely, *Daphnia spp* start reproducing sooner and produce more neonates when exposed to chemical cues released by fish [15]. It has also been shown that the presence of fish kairomones induces changes in the pattern of energy allocation, causing more energy to be directed towards reproduction at the expense of growth [16]. Besides predation risk, temperature also affects how *Daphnia spp* allocate resources to reproduction [12].

There is an extensive literature on the synergetic effects of temperature and predation in shaping life history traits [17]–[19]. But while life history responses to predation under constant temperature regimes are well known, it is less clear how *Daphnia spp* respond to predation risk under increased temperature variability. With recent global change models forecasting an increase in the variability around mean temperature [2], it is important to understand how this increased variability can affect life history responses.

The goal of this study is, therefore, to examine the effects of increased temperature variability on life history responses to different levels of predation risk. Predation may induce a response in some life history traits but not in others [14]. Hence, response to predation was examined using multiple traits. Specifically, brood size, neonate length at birth, time between broods and time to first brood were compared between individuals reared under constant and variable temperatures while exposed to high or low levels predation risk. Predation risk can also promote the evolution of inducible defenses, such as spines [20]. Therefore to complement the analysis of life history traits, the effect of predation risk on the development of defense traits was examined under both constant and variable temperatures. Finally, because of the link between temperature and predation on population dynamics [21]–[22], the synergetic effect of increased temperature variability and predation risk on population growth rate was investigated.

## Methods

The effect of increased variation in temperature in life history responses to predation was examined following the Organization for Economic and Co-operation and Development (OECD) guidelines for assessing the influence of stressors on *Daphnia magna* reproductive responses [23].

All F0 individuals used in this study were 3^rd^ brood neonates generated from *Daphnia magna* clone F [24] raised at the constant temperature of 20°C in a 16: 8 hour light: dark photoperiod in ASTM (American Society for Testing Materials) and fed with green algae *Pseudokirchneriella subcapitata*, at a concentration of 3.0×10^5^ cells mL^−1^. We decided to use this clone based on their responsiveness to fish kairomones detected in previous studies [25].

Immediately after birth, individuals were randomly allocated to either a constant (20°C; n = 30) or variable (15 to 25°C; n = 30) temperature treatment. There were no significant differences in length at birth between treatments (Table S1). Temperatures in the variable treatment varied randomly between 15 and 25**°C**. However, to avoid unrealistic temperatures, the temperature varied according to 2 sub sections - 00:00 to 08:00/18:00 to 24:00 (dawn-morning/late afternoon) and from 08:00 to 18:00 (morning and afternoon). In the dawn-morning/late afternoon section, the temperature varied between 15°C and 20°C. In the morning and afternoon section it varied between 20°C and 25°C. The daily temperature in the variable treatment varied by ∼10**°C**. Numerous studies have reported similar [26], as well as even greater [27], [28] daily variations of water temperatures. The mean temperature in the variable treatment was 19.8°C. Because the mean temperatures in the variable and in the constant treatment were similar, any effect observed in the variable treatment can be unambiguously attributed to differences in stochasticity rather than to spending different amount of time at different mean temperatures. Temperatures in the treatments were obtained using temperature controlled chambers. The temperature was checked daily.

Within each temperature treatment, 10 individuals were randomly allocated to one of three predation treatments: 1) high concentration of predator cues 2) low concentration of predator cues, or 3) substrate with no predator cues (control). We used the tropical zebrafish (*Danio rerio*), as a model vertebrate predator. Zebrafish came from laboratory cultures and we used 3-month-old individuals of similar size.

To prepare the kairomone solution, we held 20 zebrafish in 20 L aerated ASTM water for 24 hours. During this period fish were allowed to consume 400 *D. magna* of various sizes. After 24 hours, the water containing fish kairomones was filtered (0.45 mm Whatman acetate cellulose filter) and frozen at −20°C. We thawed these kairomone stock solutions 1–2 h before each medium renewal and diluted kairomones, in ASTM hard water for the three predation treatments; 1) 0.2 fish/L for high predation risk, 2) 0.05 fish/L for low predation risk and 3) ASTM for control. We renewed medium and food every other day. All individuals remained in their original conditions until the fifth generation was produced. Vials where F0's were allocated were checked daily for neonates (F1), after birth each single neonate was photographed and its total length and spine length measured using ImageJ.

The effects of increased variation of temperature on life history traits and inducible defenses in response to different levels of predation risk were analyzed using a Generalized Linear Model (GLM). Each response variable (i.e. brood size, neonate length at birth, time between broods, time to first brood and relative spine length) was analyzed separately. For all response variables the full model included two fixed factors (temperature and predation). Maternal standard length was included in the model as a covariate.

Brood size and neonate length at birth showed little departure from normality, hence they were modelled using a Gaussian error distribution. Time between broods, on the other hand, was modelled with a Poisson error distribution. Time to first brood was modelled using a negative binomial error distribution (i.e. number of days until a success [29]). Relative difference in spine length (i.e. the proportion of total length) is bounded, which makes it particularly difficult to be modeled. Because of this, the effect of increased temperature variability and predation risk in the relative difference in spine was analysed using a GLM with a Gamma error distribution and a Log link function, avoiding the problems associated to percentages being are either smaller (<20%) or bigger (>80%) [30].

For models with a known dispersion parameter (φ) (e.g. Poisson and binomial fits), Chi-squared tests are most appropriate, whereas for those with dispersion estimated by moments (e.g. Gaussian and quasi fits) the F test is best suited [31]. Also, because the value of deviance given by the Chi-squared test is analogous to the sum of squares, the proportion of total explained variance attributed to each factor of interest can be validly compared.

For each model we tested whether all factors were needed in the minimal adequate model using Akaike's Information Criterion [32]. Specifically, we calculated ΔAIC, the difference between the AIC of each model and that of the estimated best model (the model with the lowest AIC). We also calculated Akaike weights, which are estimates of the probability that each model is the best in the model set. Post-hoc multiple pairwise comparisons between groups were conducted using the multcomp package [33]. In order to estimate the strength of the association between each factor and the response variable we partitioned the explained variance of each factor within each model [34].

Finally, we investigate the interaction between increased variability in temperature and predation risk on population growth rate using the Malthusian Growth Model (eq.1).
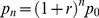
(eq.1)


The integer *p_n_* represents the number of neonates produced until time *n*, and *r* represents the average growth rate. *P*_0_ represents the starting population size (i.e. 10 individuals per treatment combination). The solution given by eq.1 is an exponential function with base (1+ *r*) raised to the power of *n*. Because the number of individuals is discrete, we assume that observations follow a Poisson distribution with the mean described as a Malthusian growth curve. Regression coefficients for the Malthusian growth rate can, therefore, be estimated using a GLM with a Poisson error distribution with a log link.

All *p*-values were subjected to Bonferroni correction. All analyses were performed using R [35].

## Results

**Figure 1:**
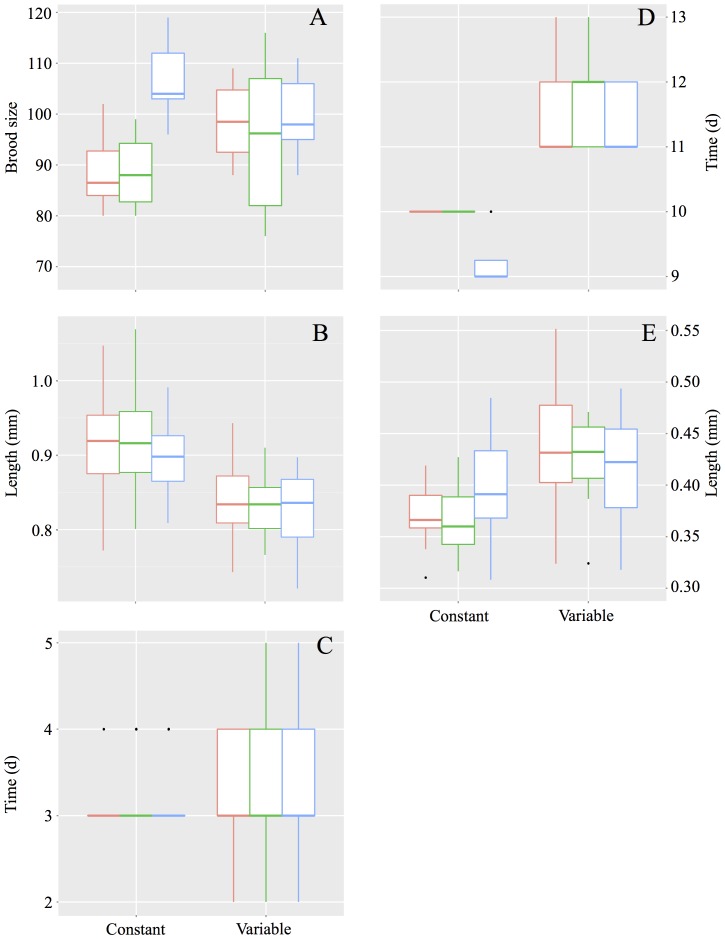
Effect of constant and variable temperatures on (A) brood size, (B) neonate length at birth, (C) time between broods, (D) time to first brood and (E) relative spine length, under no predation (Red), low predation (green) and high predation (blue) cues. Whiskers indicate 95% confidence intervals.

Our results indicate that predation risk has a significant effect on brood size (Table 1, Figure 1A). Surprisingly, we failed to detect an effect of variable temperature on brood size (Table 1, Table 2, Figure 1A). In contrast, individuals reared under constant temperature, responded to increased predation risk by producing more neonates (Table 2, Figure 1A). Analysis of total proportion of variation explained by the model revealed that temperature accounted for 21.2% and predation to 13.2% of the total model variation (Table S2).

**Table 1:** Summary of the results for Generalized Linear Model for the effect of temperature and predation on brood size, neonate length at birth, time between broods, time to first brood and relative spine length.

Response variable: brood size		df	Deviance	Test	*p*-value
Minimal adequate model ΔAIC – <0.001	Temperature	1	75.682	0.921	0.336
	Predation	2	58.490	8.596	<0.001
	Temperature * Predation	2	53.792	2.348	0.095
Response variable: Neonate length at birth					
Minimal adequate model ΔAIC – <0.001	Temperature	1	1.567	587.2	<0.001
	Predation	2	0.058	11.01	<0.001
	Temperature * Predation	2	0.012	2.384	0.092
Response variable: Time between broods					
Maximal model ΔAIC – 14.87	Temperature	1	4.03	16.1	<0.001
					
Minimal adequate model ΔAIC – <0.001					
Response variable: Time to first reproduction					
Maximal model ΔAIC – 12.3	Temperature	1	40.38	157.6	<0.001
	Predation	2	2.81	5.475	0.007
Minimal adequate model ΔAIC – <0.001					
Response variable: Relative spine length					
	Temperature	1	0.080	35.64	<0.001
Minimal adequate model ΔAIC – <0.001	Predation	2	0.029	6.566	0.011
	Temperature * Predation	2	0.019	4.267	0.025

Only best minimal adequate models are presented. The model with the lowest **Δ**AIC was selected as being the minimal adequate model.

**Table 2:** Summary of the results of post-hoc multiple pairwise comparisons after significant results obtained from the Generalized Linear Model.

	Multiple comparisons		
Response variable	Treatment	Adjusted *p*-value	
	Constant	Control vs. Low	0.999
		Control vs. High	0.005
		Low vs. High	0.007
Brood size			
	Variable	Control vs. Low	0.999
		Control vs. High	0.880
		Low vs. High	0.761
	Constant	Control vs. Low	0.319
		Control vs. High	0.041
		Low vs. High	<0.001
Neonate length at birth			
	Variable	Control vs. Low	0.924
		Control vs. High	0.149
		Low vs. High	0.822
Time between broods	Constant vs. Variable		<0.001
	Constant	Control vs. Low	0.991
		Control vs. High	0.03
		Low vs. High	0.041
Time to first reproduction			
	Variable	Control vs. Low	0.997
		Control vs. High	0.971
		Low vs. High	0.718
	Constant	Control vs. Low	0.999
		Control vs. High	0.047
		Low vs. High	0.041
Relative spine length			
	Variable	Control vs. Low	0.999
		Control vs. High	0.781
		Low vs. High	0.999

In terms of neonate length at birth, model selection included the interaction between temperature and predation as factors in the minimal adequate model (Table 1). Temperature significantly affected neonate length at birth (Table 1, Figure 1B), with smaller neonate sizes at variable temperatures (Table 2, Figure 1B). Under a constant temperature, individuals produced smaller neonates in response to increased predation risk (Table 1, Figure 1B). Under variable temperature, however, there were no significant differences in length at birth between predation treatments (Table 2, Figure 1B). Analysis of total proportion of variation explained by the model revealed that temperature accounted for 21.2% of total variation whereas predation to only 7.2% (Table S2).

Temperature, but not predation risk, significantly affected time between broods (Table 1, Figure 1C). Model selection indicated that the variation in time between broods was best explained by temperature alone (Table 1). Individuals reared at a constant temperature took less time between broods than those reared under a variable temperature regime (Table 2, Figure 1C).

There were significant differences between temperature treatments for time to first brood (Table 1, Figure 1D). Variable temperatures led to a longer time to first brood than a constant temperature (Table 2, Figure 1D). On the other hand, individuals were only able to respond to different levels of predation when at a constant temperature and not when under a variable temperature regime (Table 2, Figure 1D). Under constant temperature, time to first brood was significantly shorter when predation risk was high. Results also show that 97.4% of the total variance of the model can be attributed to temperature and only 0.8% to predation (Table S2).

Neonates produced by F0 reared at a constant temperature responded to high levels of predation risk by developing longer spines relative to their body size (Table 1, 2, Figure 1E). In contrast, neonates born to F0 reared under a variable temperature regime developed longer spines in all predation treatments (Table 2, Figure 1E). Analysis of partitioning of explained variance showed that temperature accounts for 8.47% of total variation in relative spine length and predation accounts for 0.57% (Table S2).

**Figure 2:**
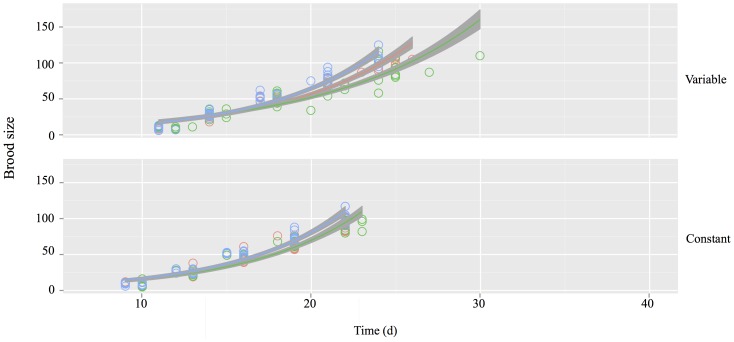
Exponential rate of population growth until the fifth brood in *Daphnia magna* reared at constant and variable temperatures and exposed to no predation (red), low predation (green) or high predation (blue) cues. Population growth rates estimated using Malthusian Growth Rate. Shaded areas indicate 95% confidence intervals.

Population growth rate trajectories as function of time were different between temperature treatments (Table 3, Figure 2). Individuals reared at a constant temperature displayed faster growth rates in comparison to those individuals reared at a variable temperature regime (Table 3, Figure 2). Growth rate was faster under high predation risk than it was at both low predation risk and under control conditions (Table 3, Figure 2).

**Table 3:** Parameter estimates for fitting regression lines for the effect of temperature and predation on growth rate using Malthusian growth rate.

Temperature	Predation	Malthusian estimate	Std. Error	95%CI	*p*-value
Constant	Control	0.09471	0.00111	0.09250–0.09688	<0.001
	Low	0.09496	0.00122	0.09254–0.09735	<0.001
	High	0.10181	0.00110	0.09963–0.10396	<0.001
Variable	Control	0.08966	0.00095	0.08778–0.09152	<0.001
	Low	0.08552	0.00095	0.08363–0.08738	<0.001
	High	0.09472	0.00101	0.09271–0.09669	<0.001

## Discussion

In this study we test the hypothesis that stress caused by increased variability in temperature leads to erroneous life history responses to predation risk. Our results support this prediction and reveal that increased temperature variability restricts life history responses to predation risk. While under a constant temperature individuals responded to high predation risk by producing more neonates at smaller sizes, and by shortening the time to produce a first brood, when temperature varied, these responses were not detected. Interestingly, predation had no effect on time between broods. This may indicate a cost-benefit ratio that exceeded the potential fitness advantages of such adjustment. Importantly, our results also show that variable temperatures have a negative effect on population growth rate and on the development of morphological predator-inducible defenses. Taken together, our results strongly support the suggestion that increased variability in temperature has far greater consequences than shifts in mean temperature, namely by leading to an inability to respond appropriately to predation risk.

Despite the intensive debate on whether global temperature is becoming more variable or not [2]–[4], [36], [37], it is acknowledged that variable temperature poses a greater threat than shifts in mean temperature [13], [38]. Our results provide strong evidence that *Daphnia magna* can detect predation risk and respond accordingly when temperatures are constant, but not when temperature varies. In most species the physiological responses to changes in temperature are often preceded by behavioural adjustments in ways that optimize fitness [39]. This process of adjustment, however, implies changes in how energy is allocated between the needs of basal rate and development of ecological traits [40]. Further, variability in temperature increases metabolic sensitivity [13]. It is conceivable that the intense conditions experienced in the variable treatment generated conflicting energetic requirements between metabolic rate and the development of predation response traits. Such a possibility is supported by suggestions that adjustments in energetic demands in response to changes in temperature may cause mismatches in traits [41], [42]. Our result confirms that increased temperature variability has a greater effect on life history responses to predation risk than mean temperatures, and lends support to hypotheses that organisms are likely to face greater difficulties in adapting to increased variability in temperature.

It has been reported that some species respond to predation risk by reducing time between broods [43]. In our case, however, time between broods was the only trait for which predation risk had no effect. The evolution of life history traits is mediated by a cost-benefit of investing in a specific trait in a given environment [6]. It is possible that the physiological cost of adjusting time between broods exceeds the potential fitness advantages of such an adjustment. Changes in basal metabolic rate in response to different temperatures have been linked to changes in time between broods [44]. Time between broods was longer in variable temperatures than under constant temperatures. Further, model selection showed that temperature was the only significant factor that best explained the variation in time between broods. This result, in combination with the link between metabolic rate and time between broods, provides a strong explanation for why we failed to detect an effect of predation risk on time between broods.

In many species the presence of predators favours the growth of ‘inducible defenses’ [20]. These are chemical and/or morphological traits that evolved as adaptations to deter predators. In the specific example of *Daphnia spp*, it has been demonstrated that chemical cues from predators induce the development of spines as morphological defenses [45]. Our results are in agreement with this study, in that they demonstrate that exposure to high, but not low, predation cues prompts the development of relatively longer spines. As with any trait, the development of inducible defenses comes at a cost [46]. The development of longer spines in response to predation risk was recorded for constant temperatures but was not observed when temperature varied. Surprisingly, regardless of the predation risk treatment, neonates developed longer spines when temperature variability was greater. This result is not consistent with the expectation that increased variation in temperature causes an energetic disequilibrium affecting the development of some traits [13]. Producing many larger offspring when the presence of predators is uncertain is likely to generate fitness costs. However, the fitness costs of producing offspring without inducible defenses when predation risk is uncertain may be fatal, especially if predators end up being present. It is possible that the extreme stress caused by variable temperature prevents individuals from assessing predation level, and given the cost-benefits of inducible defenses, it is always better to produce defenses in the absence of predators than failing to produce them under high predation.

Classical population dynamics models predict that under variable conditions and when food provisioning is constant, selection favours an increase in Malthusian growth rate, also referred to as an exponential growth [47]. Our results do not support such a prediction, as population growth rate was slowest when temperature was variable. It has also been suggested that high levels of predation risk are expected to suppress breeding [48]. Again, we failed to detect an effect of predation on breeding suppression. Population growth rate was faster under high predation cues than under low predation cues or in the control. This result, although unexpected, is in line with previous studies on population growth rate (eg. [25]). We used zebrafish to produce kairomones, and zebrafish prey upon all size classes of *Daphnia spp*. Effects of predation risk in suppressing breeding have been reported for when predators prey upon a particular size class (smaller sized) [48], [49]. Given this, the absence of size-specific predation risk may explain why population growth rate was faster under high predation risk. It is also possible that an increase in population growth rate is an intrinsic consequence of an earlier time to first reproduction and greater number of neonates produced.

Our work adds to the body of knowledge regarding the significant role of predators in shaping all levels of the organizational system of a prey population [9]. Demographic selection caused by predator-induced prey mortality, may pass along the entire network causing a cascade effect within the trophic web [50]. The consequences of failing to respond to predation go, therefore, beyond the direct effects on the target species and may result in shifts at the community, and even the ecosystem, level. Our results provide conclusive evidence that increased variability in temperature disrupts the ability to respond to predation risk. Moreover, there are suggestions that species may be more vulnerable to variability in temperature than upward shifts in mean temperature [13], [38]. Our results empirically support such concerns and highlight the importance of considering interactions among multiple stressors on life history responses.

## Supporting Information

Table S1 **Proportion of total variance explained by the model attributed to Temperature and Predation, for brood size, neonate length at birth, time between broods, time to first reproduction and relative spine length.**
(DOCX)

Table S2 **Means and standard deviation of maternal length at brood 1, 2, 3, 4 and 5.**
(DOCX)
