# Supplementary material for: Predation Life History Responses to Increased Temperature Variability
Source: PLoS One. 2014 Sep 24;9(9):e107971. doi: 10.1371/journal.pone.0107971 (PMC4176018; doi:10.1371/journal.pone.0107971)
Supplement: Table S2 — Means and standard deviation of maternal length at brood 1, 2, 3, 4 and 5. (DOCX) [file pone.0107971.s002.docx]

| Response variable | Sources of variation | | |
| --- | --- | --- | --- |
|  | Temperature | Predation | Temperature * Predation |
|  |  |  |  |
| Brood size | 21.1 | 14.6 | 13.2 |
| Neonate length at birth | 21.1 | 7.23 | 10 |
| Time between broods | 2.69 |  |  |
| Time to first brood | 58.1 | 3.36 |  |
| Relative spin length | 8.47 | 0.57 | 0.53 |

Table S2 – Proportion of total variance explained by the model attributed to temperature and predation, for brood size, neonate length at birth, time between broods, time to first reproduction and relative spine length.
